# Influence and Sensitivity of Temperature and Microstructure on the Fluctuation of Creep Properties in Ni-Base Superalloy

**DOI:** 10.3390/ma13214758

**Published:** 2020-10-24

**Authors:** Zhihao Yao, Biao Zhou, Kaijun Yao, Hongying Wang, Jianxin Dong, Theresa Davey

**Affiliations:** 1 High Temperature Materials Research Labs, School of Material Science and Engineering, University of Science and Technology Beijing, Beijing 100083, China; g20188466@xs.ustb.edu.cn (B.Z.); s20190397@xs.ustb.edu.cn (K.Y.); s20180366@xs.ustb.edu.cn (H.W.); jxdong@ustb.edu.cn (J.D.); 2 Fracture and Reliability Research Institute, Tohoku University, 6-6-11-716 Aramakiaza-Aoba, Aoba-ku, Sendai 980-8579, Japan; theresa@tohoku.ac.jp

**Keywords:** Ni-based superalloy, solution treatment, gamma prime (γ′) phases, sensitivity zone, creep residual strain

## Abstract

In this work, the sensitivity zone of microstructure and temperature for precipitation-strengthened nickel-based superalloys, used for turbine applications in aero-engines, has been firstly established. Heat treatment experiments with different solution temperatures were carried out. The microstructure evolution and creep residual strain sensitivity, low cycle fatigue properties, and tensile properties are analyzed, and the essential reason for the fluctuation of the mechanical properties of nickel-based superalloys was revealed. The main results obtained are as follows: following subsolvus solution heat treatment with a temperature of 1020 °C, samples have a high primary γ′_I_ phase content, which is beneficial to low creep residual strain. Above the supersolvus solution temperature of 1040 °C, the creep residual strain value and low cycle fatigue performance fluctuate significantly. The essential reason for the dramatic fluctuation of performance is the presence of γ′ phases in different sizes and quantities, especially following the solution heat treatment in the temperature-sensitive zone of the γ′_I_ phase, which is likely to cause huge fluctuations in the microstructure of tertiary γ′_III_ phases. A zone of particular sensitivity in terms of temperature and microstructure for the γ′_I_ phase is proposed. The range of suitable solution temperatures are discussed. In order to maintain stable mechanical properties without large fluctuations, the influence of the sensitivity within this temperature and microstructure zone on the γ′ phase should be considered.

## 1. Introduction

The nickel-based superalloy WASPALOY (UNS N07001, similar to GH4738 alloy) is a nickel- base, age hardenable superalloy with excellent high-temperature strength and good corrosion resistance, it is commonly used as the material for engine turbine disks of aircrafts and in power-plant turbines because of its excellent resistance against high temperature creep and fatigue, where these properties are derived from the certain volume fractions of the γ′ strengthening phase (an L1_2_-ordered Ni_3_Al/Ti precipitate) and controllable grain sizes [1,2,3,4]. The nickel-based superalloy typically exhibits a trimodal distribution of γ′ phases. During processing, the alloy will be consolidated by deformation (such as forging and extrusion), forming primary γ′ phases (γ′_I_ phases) and affecting the volume fraction and precipitate size, which will typically be between 100 and 200 nm [2]. The primary γ′ precipitate has the role of restricting grain boundary migration during subsolvus solution heat treatments. Subsequent heat treatments can be used to promote the formation of two further intragranular precipitate populations, denoted as secondary and tertiary γ′ phases (γ′_II_ and γ′_III_ phases), with diameters typically between 50 and 100 nm and 5 and 50 nm, respectively [4,5].

Generally, the alloy used relies on carefully tailored compositions and heat-treatment schedules that result principally in a microstructure constituting of the γ′ phases [6,7,8,9,10,11,12]. It is typically subjected to three-stage supersolvus or subsolvus standard heat treatment for the alloy as follows. (1) Solution and air quenching, which will mainly control the grain sizes during solution treatment, while both quenching after solution treatment and aging determines the size, morphologies, and distribution of γ′ precipitates. The quenching process plays a much more important role in superalloy heat treatment than aging because it significantly controls the nucleation and growth kinetics of γ′ precipitates and sets the stage for subsequent aging. It is found that the higher the cooling rate, the higher the strength will be. (2) Stabilization and air cooling, which mainly affect M_23_C_6_ (are Cr-rich fine secondary carbides, M mainly refers to Cr, NI, Mo and Fe elements) carbides. (3) Aging and air cooling, which precipitate a refined dispersion of γ′ phases.

Once the subsolvus heat treatment I is conducted as 996–1038 °C/4 h/air cooling (AC) or oil cooling (OC)) + 843 °C/4 h/AC + 760 °C/16 h/AC, the alloy possesses good tensile strength and fatigue resistance at room and elevated temperature where its γ′ phases show bimodal sizes and spherical distribution in matrix; otherwise, if we need the alloy to obtain the best creep or stress rupture properties, the supersolvus heat treatment II should be conducted as 1038–1080 °C/4 h/AC + 843 °C/24 h/AC + 760 °C /16 h/AC, which results in an alloy with uniform and fine γ′ phases and coarse grains [12].

As documented in numerous studies, the creep behavior is closely related to the morphology and size of the precipitates and greatly impacts the mechanical properties [1,13,14,15,16,17]. Indeed, the optimization of the requisite properties is achieved by a combination of grain size control, distribution of minority phases such as carbides and borides and most importantly a multisized distribution of γ′ phases. Interaction is influenced by the lattice parameter misfit between the ordered precipitate and the matrix, combined with the size and volume fraction of the precipitates that have been reported to affect properties. Depending on heat treatment and the γ/γ′ lattice mismatch, the morphology of the secondary γ′ phases evolves from initial spheres, through cubes and octocubes to more complex structures. Higher lattice mismatches lead to a faster coherency loss and a transition to more complex structures during heat treatments. Slower cooling rates or isothermal heat treatments at high temperatures lead to more complex structures [18,19,20,21,22].

Several authors have pointed out that as the alloy’s dissolution temperature range for γ′ phases can be from 968 to 1059 °C in standard chemical composition ranges, it will significantly affect the volume of γ′ phases with different solution temperature [23,24,25,26]. With increasing Al and Ti content, the dissolution temperature increases while the essential character of change is the difference of key elements, specifically Al and Ti, which contribute to the formation of γ′ phases. Large primary γ′ phases pin the grain boundaries and thus stabilize the microstructure, while the finely dispersed secondary and tertiary γ′ phases are responsible for the high strength of the material. The precipitation occurs in three stages: nucleation, growth, and coarsening [27,28,29,30,31]. Since precipitation is a diffusion-controlled process, the nucleation and growth mechanisms are strongly dependent on the temperature evolving continuously during cooling. The nucleation and growth of tertiary γ′_III_ phases can be explained by the competition between increasing supersaturation due to continuous cooling and decreasing supersaturation due to the formation of secondary γ′_II_ phases in the matrix.

The factors controlling the creep performance of the disc alloys remain largely unexplored, and the formation mechanisms contributing to performance sensitivity have not been studied in detail, but it has been highly suspected that this mechanism was correlated with the presence of γ′_I_ phases [32,33,34,35,36]. In particular, creep properties are susceptible to variation when heat treatment occurs near the γ′_I_ phase solvus temperature due to the combination of different classes of γ′ precipitates. Therefore, the first objective in the study will be to explore the relationship between the content of Al and Ti, γ′ phases, and solution temperature [37,38,39,40]. In recent progress, USTB (University of Science and Technology) has developed China’s largest diameter WASPALOY disk for heavy duty gas turbines [41]. The grain size distribution can be controlled by accurate deformation parameter design, while its precipitates need deeper study.

In this study, a mechanism of temperature sensitivity was proposed for the near γ′-solvus temperature heat treatment that is required for the optimization of alloy production. This study was conducted on three alloys with differing content of Al + Ti from 4.4 to 4.48 pct (percentage, %), while their composition is all within the range of WASPALOY, which undergo heat treatment at different solution temperatures and aging. Furthermore, the creep, tensile, and fatigue properties of the samples have been tested. We found that a severe fluctuation of mechanical properties occurs in a particular temperature range spanning a zone of increased sensitivity to the γ′_I_ phase. By analyzing the morphology and volume of the γ′_I_ phases, the evolution and creep sensitivity of the γ′_I_ phases were studied. Finally, the key control factors for mechanical properties were proposed, and a model between the Al + Ti content, solution temperature, and γ′_I_ phase was established, which will lay the foundation for further research and development of the alloy. Further, it will be extremely important that the performance and stability of the alloy can be easily controlled, and this will provide an important topic for future research.

## 2. Materials and Methods

All experimental WASPALOY samples were prepared by same standard process and followed vacuum induction melting (VIM), vacuum arc remelting (VAR), and homogenization and rolling after different Al + Ti composition design. The cylindrical alloy specimens used in this study were derived from rolled bars with a diameter of 48 mm that were cut down by wire electrical discharge machining. The detailed chemical composition of the alloys tested by Inductively Coupled Plasma-Atomic Emission Spectrometer (ICP-AES, the equipment made by Agilent in US, the test standard followed by ASTM E2594-09, which it is a Standard Test Method for Analysis of Nickel Alloys by Inductively Coupled Plasma Atomic Emission Spectrometry; Performance-Based Method, reapproved 2014) are shown in Table 1. It was clear that three alloys have remarkable difference in Al + Ti from 4.39 to 4.48. Then specimens underwent a three steps heat treatment: solution + stabilization + aging treated as follows: 1020 °C/4 h/AC, OC + 845 °C/4 h/AC + 760 °C/16 h/AC, and 1040 °C/4 h/AC + 845 °C/4 h/AC + 760 °C /16 h/AC. In order to reduce the influence of external interference factors on variations in microstructure and properties, such as variations in furnace temperature and nonuniformity of temperature within the furnace, all samples were carefully heat treated at the same heat furnace (Produced by CTJZH, Tianjin, China) and cut from same position along the length of the bars, etc. Hence, it is credible to quantitatively assess property variability reported in the present work within solution-temperature regime, which may be expected to give rise to such variability.

**Figure 1:**
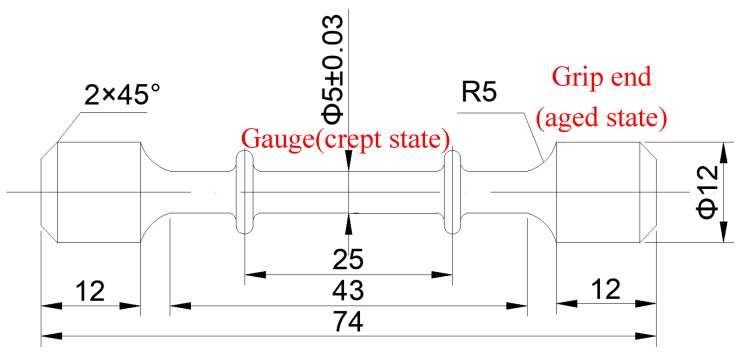
The schematic diagram of the creep samples.

After heat treatment, cylindrical specimens were processed into creep samples. The schematic diagram of the creep samples is shown in Figure 1. The gauge length and diameter of creep samples were 25 and 5 mm, respectively. Each sample was stretched under constant stress and temperature in air by means of an RMT-D10 electronic high-temperature creep testing machine (Produced by SUST, Zhuhai, China). The samples were creep tested at 750 °C with stress of 400 MPa and load held for 60 h, and then, their elongation (also called creep residual strain, CRS for short) was examined without fracture failure.

Besides the standard tensile tests at room and elevated temperature of 750 °C, a low cycle fatigue experiment was also conducted for comparison in the study, under conditions of 700 °C, strain amplitude 0.5%, stress ratio R = 0.1. In order to obtain precise results, property data was collected for each test condition by performing experiments on two samples of each type in parallel (e.g., specimen 1 abbreviated to s1).

In addition, to investigate the microstructure evolution in samples during creep at elevated temperature, the grip end of the creep samples (shown in Figure 1 and with a diameter of 12 mm) were electrolytically polished and extracted by electrolyte solution after each experiment. The microstructure evolution of the grip end (which underwent no stress and having undergone isothermal treatment) were also examined using Zeiss SUPRA 55 field emission gun FSEM (Made by Zeiss, Obercochen, Germany) operating at 5–15 kV. Prior to the studies, the samples underwent traditional metallographic procedures using SiC paper to grind from 60 to 7000 grits, followed by mechanical polishing with 2.5 and 0.5 μm diamond pastes. For observation of precipitates, samples were electrolytically polished in a solution of 20% H_2_SO_4_ + 80% CH_3_OH (Methanol) with a voltage of 15 V for 3 s and then electrolytically etched in a reagent of 150 mL H_3_PO_4_ + 10 mL H_2_SO_4_ + 15 g CrO_3_. Additionally, precipitates sizes were measured by using the image analysis software Image Pro. Plus 6.0.

## 3. Mechanical Properties

### 3.1. Creep Residual Strain

**Figure 2:**
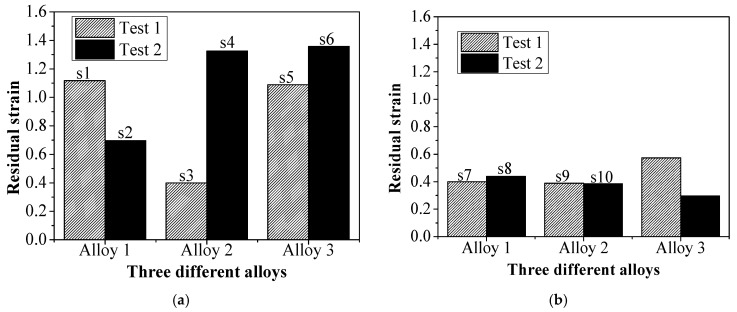
The residual creep strain of 750 °C/400 MPa for 60 h after two different heat treatments solution temperature at (**a**) 1040 °C and (**b**) 1020 °C.

The CRS results are very interesting for the three alloys after both kinds of heat treatments. As shown in Figure 2, in general, (1) after the solution heat treatment at 1040 °C, the residual strain values of the two samples vary more, even though they were from the same alloy bar. In contrast, the solution heat treatment at 1020 °C has less difference between samples; (2) after careful observation, it was found that after the solution heat treatment at 1020 °C, although the fluctuation of the residual strain is small, there is the largest difference between the two samples of Alloy3; (3) at the same time, the study found that after solution heat treatment at 1040 °C, the two samples of Alloy1 and Alloy2 have a large deviation, while in the same tests, Alloy3 has a smaller deviation. An in-depth study is needed to reveal what causes the fluctuation of this creep residual strain.

The above experimental results showed that the CRS had large fluctuations, despite only slight changes to the composition in the range of the alloy after the same solution treatment. This would inevitably have a negative effect on the stability control of performance. How to accurately control the stability is of great significance. Therefore, it is necessary to analyze the mechanism of fluctuations in the creep residual strain of the alloy.

### 3.2. Low Cycle Fatigue

**Figure 3:**
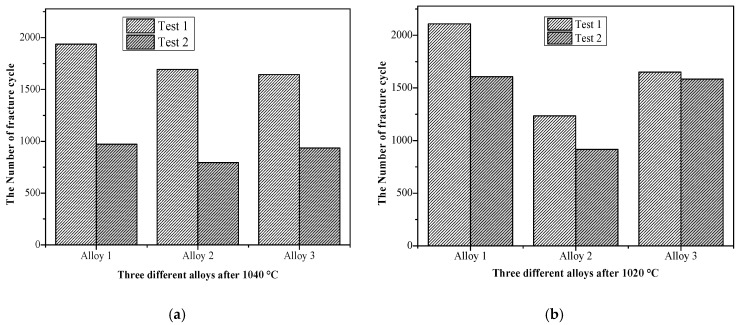
The low cycle fatigue properties at 700 °C/strain amplitude 0.5%/stress ratio R = 0.1 after two different heat treatments solution temperature at (**a**) 1040 °C and (**b**) 1020 °C.

The trends of the low cycle fatigue properties were similar to the above test results of creep residual strain. It can be seen from Figure 3a that after solution heat treatment at 1020 °C, the ratio of the smaller number to the larger number of fatigue fracture cycles between the two parallel samples are: 0.76, 0.74, and 0.96. As can be seen from Figure 3b, after solution heat treatment at 1040 °C, the ratios of the tested values are 0.50, 0.47, and 0.57, respectively. It can be seen that the fatigue data after solution treatment at 1020 °C had less fluctuation compared to the performance fluctuation after solution treatment at 1040 °C. In addition, the number of fatigue fracture cycles after solution heat treatment at 1020 °C was higher than at 1040 °C.

### 3.3. The Tensile Yield Strength

As shown in Table 2, the alloys were subjected to two solution heat treatments: Heat No. 1: 1020 °C/4 h/AC + 845 °C/4 h/AC + 760 °C/16 h/AC and Heat No. 2: 1020 °C/4 h/OC + 845 °C/4 h/AC + 760 °C/16 h/AC. These tests showed that the tensile yield strength after 1020 °C solution heat treatment + oil cooling was higher than after the same heat treatment followed by air cooling. It was also seen that as the Al + Ti content increased, the alloy′s tensile strength increased. The trends for the yield strength were reasonable based on the compositions of the three alloys [1,39].

From the analysis of the creep residual strain, the low cycle fatigue fracture, and the room temperature tensile yield strength data, the following preliminary results can be shown: (1) A small variation in Al + Ti content has a greater impact on properties and causes fluctuations. (2) After solution heat treatment at 1020 °C, the value of creep residual strain was low with small fluctuations, and after solution heat treatment at 1040 °C, the creep residual strain value was high and the fluctuations were large. When the content of Al + Ti in the alloy was high, the variation of creep residual strain was large after solution heat treatment at 1020 °C and small after solution heat treatment at 1040 °C. (3) The impact of solution temperature on the low cycle fatigue fracture was not obvious, but the chemical composition appears to have a greater impact on performance. (4) After solution heat treatment at 1020 °C and oil cooling, the tensile yield strength at room temperature was higher; the increase in Al + Ti content was beneficial in increasing the tensile strength.

## 4. Microstructure Examination

### 4.1. Creep Sensitivity After Solution Temperature at 1040 °C

**Figure 4:**
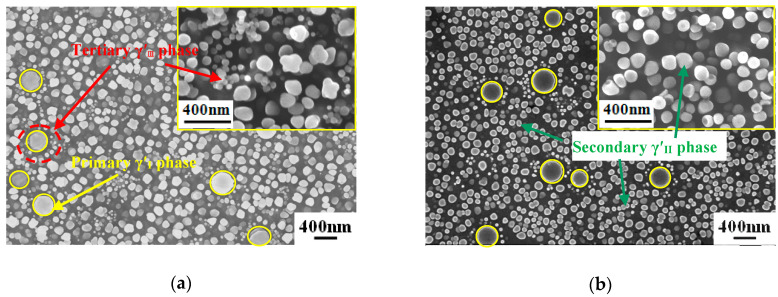
The distribution of γ′ phases in Alloy1 after solution heat treatment at 1040 °C. (**a**) specimen2 and (**b**) specimen1.

Figure 4 shows the morphology of the γ′ phases from the Field Emission Scanning Electron Microscope (FESEM) in both specimens of Alloy1 after creep tests. It can be observed that there is no obvious difference in the strengthening phases as a whole. It can be seen that three size classes of precipitates are present in the matrix. These are conveniently referred to as the primary γ′_I_ (marked with yellow, 300–380 nm), secondary γ′_II_ (marked with green, 80–200 nm), and tertiary γ′_III_ phases (marked with red, 10–40 nm), and their sizes and volume fractions vary depending upon the alloy compositions and the particular heat treatment. Remarkably, the γ′_III_ phases mostly precipitate around primary γ′_I_ phases.

However, further observation of Figure 4a,b shows subtle differences between the γ′ phase size in the two specimens (labelled s1 and s2 in Figure 3) of Alloy1. That is, specimen1 has more secondary γ′_II_ and tertiary γ′_III_ phases than specimen2. Figure 4a shows the enlarged morphology. The creep residual strain value of 0.696 for specimen2 is lower than 1.120 for specimen1, both of Alloy1. It can be seen that for a given solution temperature in a heat treatment process, the total volume of γ′ phases depends on the composition of the alloy. The more the finely dispersed phases that precipitate, the greater the contribution to creep properties of the alloy. This is probably the main reason for the difference in the creep residual strain values of specimen1 and specimen2.

**Figure 5:**
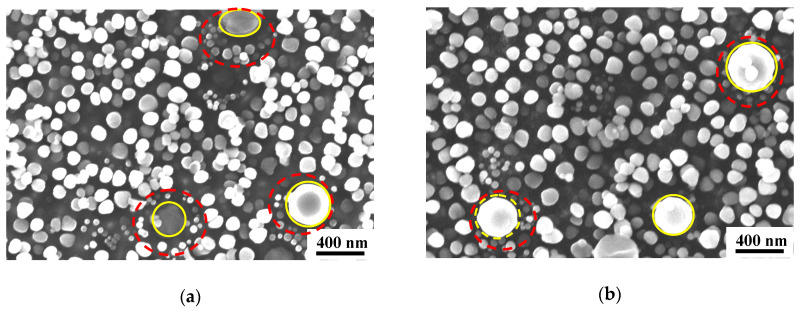
The distribution of γ′ phases in Alloy2 after solution heat treatment at 1040 °C. (**a**) specimen3 and (**b**) specimen4.

As shown in Figure 5, specimen4 (as labelled in Figure 3) has more tertiary γ′_III_ phases than specimen3 (both Alloy2). The creep residual strain value of 0.399 for specimen3 is lower than 1.318 in specimen4. It is obvious that more tertiary γ′_III_ phases around the primary γ′_I_ phases contribute to a lower creep residual strain value, even though both specimens underwent the same heat treatment. From the experimental results shown in Figure 2, it is consistently seen that the creep residual strain value is lower after heat treatment with solution temperature 1020 °C compared to 1040 °C.

**Figure 6:**
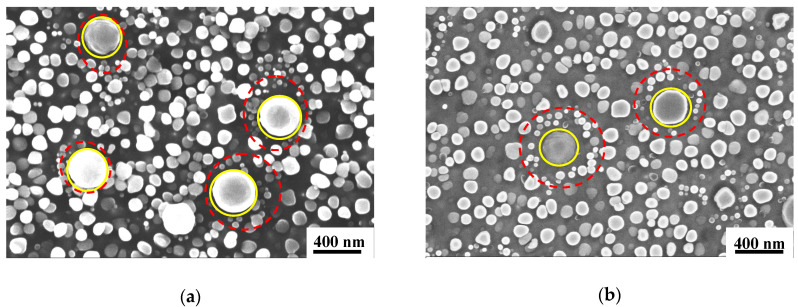
The distribution of γ′ phases in Alloy3 after solution heat treatment at 1040 °C. (**a**) specimen6 and (**b**) specimen5.

Figure 6 shows the distribution of γ′ phases in specimen5 and specimen6 (as labelled in Figure 3) of Alloy3 after solution heat treatment at 1040 °C, in which the creep residual strain values are 1.092 and 1.362, respectively. We can see the γ′ phases are similar, and both specimens include three classes of γ′ phases, in particular, a large amount of tertiary γ′_III_ phases appears around the primary γ′_I_ phases. The dissolution temperature of γ′ phases in Alloy3 is higher due to its higher Al + Ti content, so the primary γ′_I_ phases remain even following heat treatment with solution temperature of 1040 °C. The increasing amount of tertiary γ′_III_ phases make the variations in the value of creep residual strain decrease.

### 4.2. Creep Performance After Solution Temperature at 1020 °C

**Figure 7:**
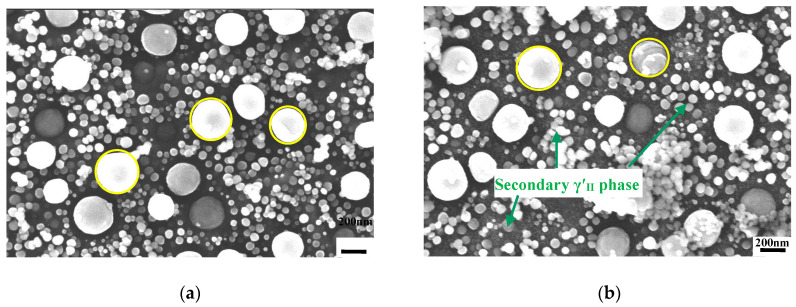
The distribution of γ′ phases in Alloy1 after solution heat treatment at 1020 °C. (**a**) specimen7 and (**b**) specimen8.

Figure 7a,b shows the distribution of γ′ phases in specimen7 and specimen8 of Alloy1 (as labelled in in Figure 2b) after solution heat treatment at 1020 °C. Both specimens have more primary γ′_I_ phases than seen after solution heat treatment at 1040 °C. As there is a depleted area instead of tertiary γ′_III_ phases around primary γ′_I_ phases, the microstructure mainly includes primary γ′_I_ phases and secondary γ′_II_ phases. From Figure 1, we can see that the creep residual strain values of specimen7 and specimen8 are 0.386 and 0.389, respectively. It is clear that the creep residual strain value fluctuates a little after solution heat treatment at 1020 °C, and the creep residual strain values are lower than at 1040 °C.

It is known that the tensile properties of alloys are mainly affected by grain size and volume of precipitates. However, from the above analysis, it can be seen that the difference in tensile properties after solution heat treatment at 1020 or 1040 °C is mainly due to obvious differences in the γ′ phases.

### 4.3. The Influence of Cooling Rate on Microstructure

**Figure 8:**
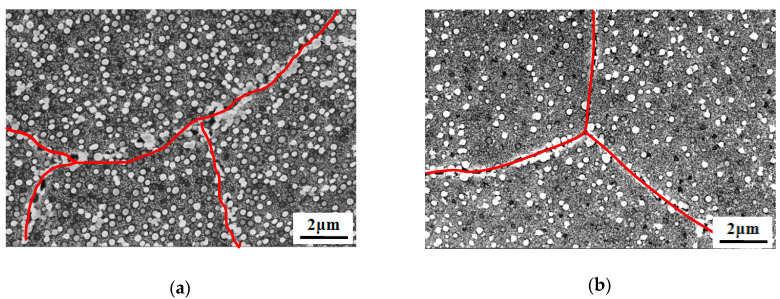
The distribution of γ′ phases in Alloy3 after solution heat treatment at 1020 °C and (**a**) air cooling and (**b**) oil cooling.

Figure 8a,b shows how the distribution of γ′ phases is dependent on cooling rate after solution heat treatment at 1020 °C. It is clearly seen that the alloy has more primary γ′_I_ phases after air cooling than oil cooling, because they failed to dissolve into the matrix in time. That is to say, the faster the cooling rate from the solution heat treatment, the fewer primary γ′_I_ phases are. Because the heat treatment involves subsequent 845 °C/4 h/AC + 760 °C/16 h/AC, the precipitation of finer γ′ phases is dominant after solution heat treatment plus oil cooling.

It was also found that the tensile yield strength at room temperature is higher after solution heat treatment at 1020 °C plus oil cooling than plus air cooling. In Table 2, it is shown that the tensile yield strengths are 909.3, 924.6, and 960.0 MPa after solution heat treatment plus oil cooling for Alloy1, Alloy2, and Alloy3, respectively, and correspondingly are 780.1, 769.9, and 810.2 MPa following the same heat treatment plus air cooling. In summary, this explains how the cooling rate has an important effect on the microstructure and its properties. The more finer phases that are present, the higher the tensile yield strength is for the alloy. Additionally, both specimens have similar grain boundaries (marked with red lines in Figure 8) after AC and OC heat treatment, and there is very little carbide precipitation, as shown by the red line marking the grain boundary locations of the carbide precipitates in Figure 8, so this can be seen to have a relatively negligible effect on the influence of properties given the change of phases.

Through comparative analysis, it can be seen that the essential reason for higher tensile properties after oil cooling following solution heat treatment at 1020 °C is that the amount of γ′_I_ phases decreases, which leads to an increase in the number of dispersed γ′_II_ phases and even γ′_III_ phases. In short, the main reason for the difference in tensile properties is that the increased cooling rate reduces the amount of γ′_I_ phases, while the total amount of γ′ phases is kept constant after solution heat treatment + double aging. Therefore, the number of γ′_II_ and γ′_III_ phases is bound to increase, which furthermore leads to the increased yield strength. In view of the many factors that affect the γ′ phases, this will be discussed in terms of the following main factors: (1) the amount of Al + Ti, (2) the solution temperature, and (3) the cooling rate.

## 5. Discussion

### 5.1. The Evolution of γ′ Phases with Solution Temperature

**Figure 9:**
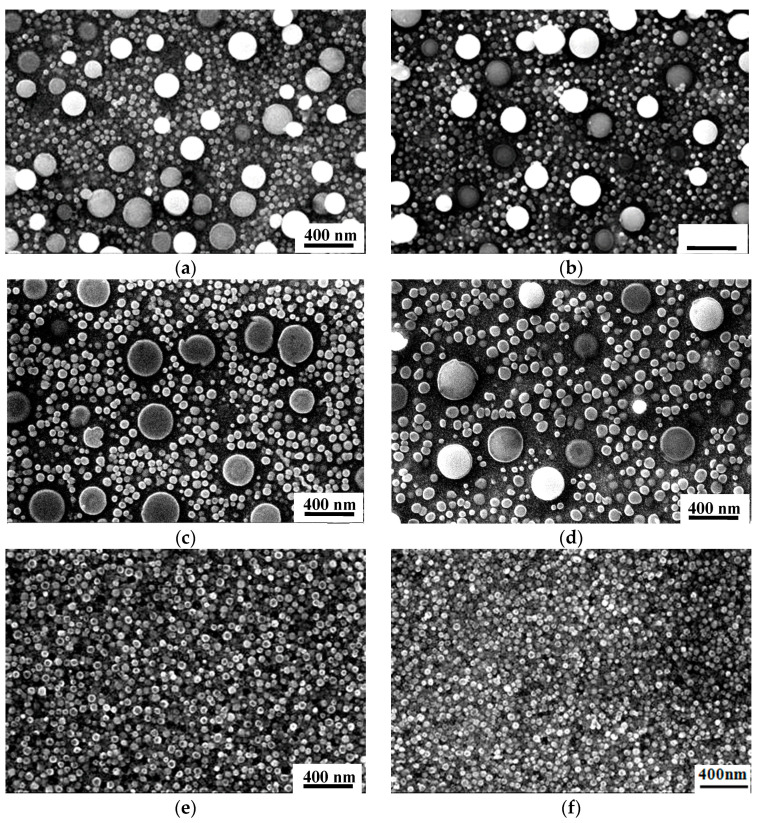
The distribution of γ′ phase in Alloy2 following solution heat treatment at varying temperatures. (**a**) 1000 °C, (**b**) 1010 °C, (**c**) 1020 °C, (**d**) 1040 °C, (**e**) 1060 °C, and (**f**) 1080 °C.

In order to systematically study the relationship between the solution heat treatment temperature and the γ′ phases, the following solution heat treatment experiment was performed on Alloy2: the solution heat treatment was performed at 1000, 1010, 1020, 1040, 1060, and 1080 °C for holding times of 4 h then air cooling plus double aging as 845 °C/4 h/AC + 760 °C/16 h/AC. Figure 9 shows the distribution of γ′ phases following heat treatment at different solution temperatures.

It can be seen from Figure 9 that the most obvious difference following the different solution heat treatment processes is the evolution of the characteristics of the γ′ phases. The γ′ phases appear with different morphologies and sizes, including the primary γ′_I_ phases, secondary γ′_II_ phases, and tertiary γ′_III_ phases. As the solution temperature increases, the number of primary γ′_I_ phases decreases, but the size of the undissolved primary γ′_I_ phases increases. When solution temperature is above the turning point temperature of γ′ phases around 1040 °C, the γ′ phases become unusually sensitive and complex, intensifying the dynamic dissolution and precipitation processes. After treatment at a solution temperature of 1060 °C, all primary γ′_I_ phases are completely dissolved in the matrix, the subsequent precipitated γ′ phases show a uniform distribution during aging.

**Figure 10:**
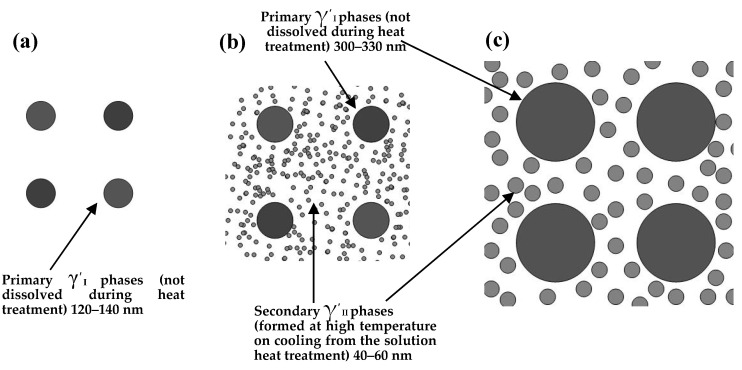
Schematic diagram of the evolution of γ′ phases: (**a**) forging state, (**b**) heat treated at solution temperature 1000 °C, and (**c**) heat treated at solution temperature 1020 °C.

Figure 10 shows a schematic diagram of the evolution of the γ′ phases. It can be seen that the alloy has uniform size γ′_I_ phase (about 120–140 nm) in the as hot forged state, whereas the other γ′ phases are completely dissolved in the matrix during the hot deformation process. When it is heat treated at solution temperature of 1000 °C, the primary γ′_I_ phases grow to about 300–330 nm, and some secondary γ′_II_ phases (40–60 nm) are precipitated during the cooling process. When the solution temperature is increased to 1020 or 1040 °C, the microstructure changes significantly. After solution heat treatment at 1020 °C, the primary γ′_I_ phase precipitates grow to about 330 nm. After heat treatment at 1040 °C, most of the primary γ′_I_ phase dissolves into the matrix, whereas the remaining the primary γ′_I_ phase precipitates grow to about 400 nm. During this period, a large number of fine tertiary γ′_III_ phases precipitates around the primary γ′_I_ phases, whereas no tertiary γ′_III_ phases precipitate around the secondary γ′_II_ phases. Any secondary and tertiary gamma primes that are observed likely develops during cooling or aging and results from supersaturation that develops during cooling or which is retained at the beginning of aging. The temporal and spatial variation in supersaturation and its effect on both nucleation and growth is the key to determine how the secondary and tertiary precipitates form [42]. The variation of the creep residual strain value is mainly due to the sensitivity to varying precipitation of tertiary γ′_III_ phases that occurs at 1040 °C.

**Figure 11:**
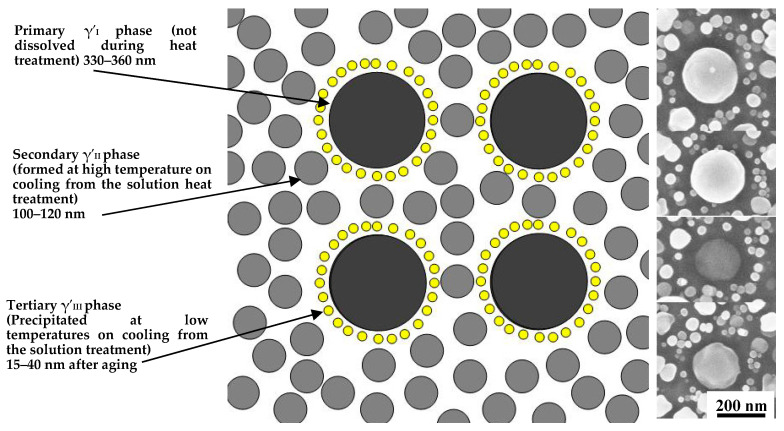
Schematic illustration of the γ′ phases after heat treatment at solution temperature 1040 °C and double aging.

In order to more clearly observe the distribution of the γ′ phases, Figure 11 shows a partial enlarged view of the γ′ phases and schematic illustration of the γ′ phases after heat treatment at solution temperature of 1040 °C. It can be clearly seen that the primary γ′_I_ phases are tightly surrounded by tertiary γ′_III_ phases. The reason could also be clarified that the higher (near-solvus) solution temperature results in a more highly enriched matrix. Cooling of such a matrix at a given rate will tend to produce coarser secondary gamma prime (due to nucleation and growth at higher temperatures), which, in turn, may result in local supersaturations adjacent to the remaining primary gamma prime that are great enough to lead to nucleation of tertiary gamma prime phases.

The alloy will possess fine tertiary γ′_III_ phases precipitated around the primary γ′_I_ phase after heat treatment at 1040 °C. These fine tertiary γ′_III_ phases will inevitably affect the value of the creep residual strain. Therefore, the fine phases should be fully considered during heat treatment at 1040 °C in order to achieve production with stable properties.

**Figure 12:**
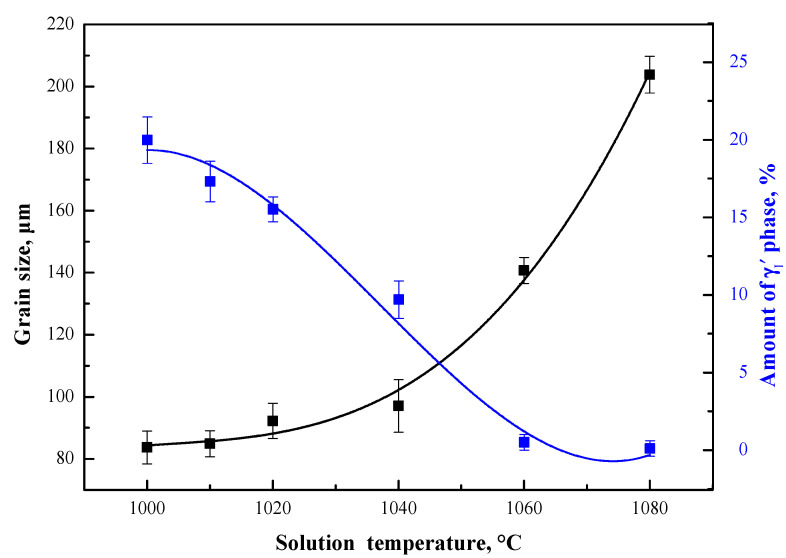
Effect of solution temperature on grain size and the amount of γ′_I_ phases.

Figure 12 shows the effect of solution temperature on the grain size and the amount of primary γ′_I_ phases. It can be seen from Figure 12 that when the solution temperature is below 1040 °C, the γ′ phases have a strong influence on grain growth by retarding the migration of grain boundaries. Up to 1040 °C, the grains grow slowly and their size is around 90 μm; if the solution temperature is increased to 1040 °C, which is higher than the starting temperature of γ′ phase dissolution, the pinning effect of the γ′ phase rapidly weakens and the grains grow quickly. At the same time, the change of solution temperature also affects the morphology distribution of the γ′ phase, where the number of γ′_I_ phase precipitates gradually decreases as the solution temperature increases.

In particular, it is worth mentioning that heat treatment at around 1040 °C is probably the transition temperature point from the stable existence to drastic changes in the alloy. Not only does the evolution of the γ′ phase significantly change but also the growth trend of grain size has an inflection point. Of course, the range of this temperature sensitivity is directly related to the composition (especially the content of Al + Ti), where the transition temperature increases with increasing Al + Ti content. Therefore, it can be speculated that the size and number of γ′ phases will be affected by both composition and heat treatment solution temperature, especially close to the transition temperature.

**Figure 13:**
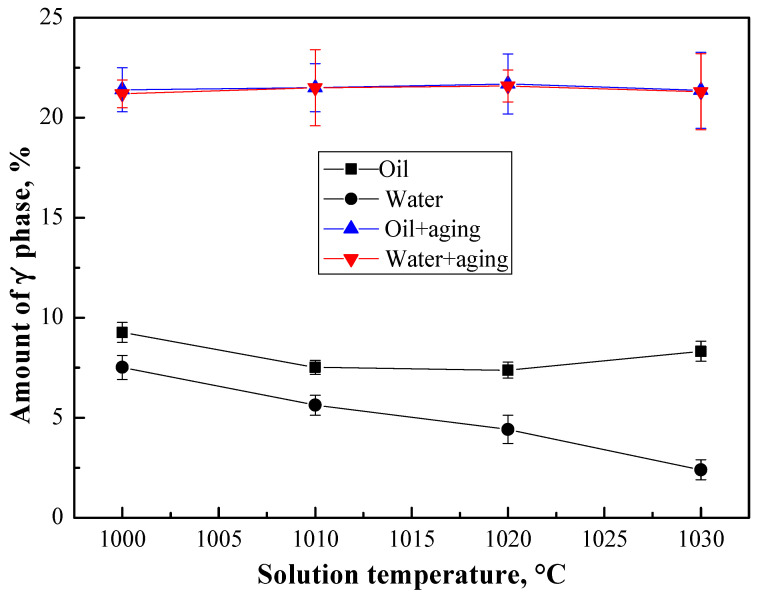
The influence of cooling rate on the amount of γ′ phase [4].

In order to further study the relationship between solution temperature and the total amount of γ′ phase, the content of the γ′ phase was analyzed following different heat treatment solution temperatures and cooling conditions. Then the samples with the different cooling conditions described above were subjected to the standard double aging treatment, and their γ′ phase content was analyzed (see Figure 13). It can be seen that the amount of the γ′ phase precipitated varies during different cooling rate conditions, but after the subsequent double aging treatment, the total amount of γ′ phase is the same.

**Figure 14:**
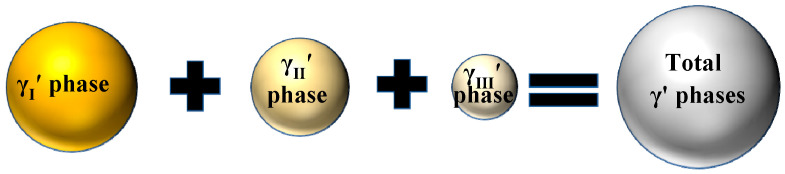
Schematic diagram of the connection between the total amount of γ′ phases and the amount of each morphology of γ′ phases.

This means that the total amount of γ′ phases is determined by the alloy composition. Therefore, it can be inferred that once the alloy composition is determined, after certain heat treatments have been performed, the total amount of γ′ phases is constant. The size distribution of γ′ phases depends on the heat treatment solution temperature and aging treatment. Figure 14 schematically shows the relationship between the different morphologies of γ′ phases, i.e., given the premise that the total amount of γ′ phase is constant (if the composition is fixed), if the amount of γ′_I_ phases decreases, it will inevitably lead to an increase in γ′_II_ and γ′_III_ phases. Through the above experiments, it was found that the number of the primary γ′_I_ phases, secondary γ′_II_ phases, and tertiary γ′_III_ phases has a mutually transformational relationship.

In short, the temperature where there is a sharp change in the dissolution of the γ′_I_ phase is determined by the composition, and it significantly affects the size morphology of the γ′ phase. Therefore, we can control the γ′ phases near this temperature to reduce fluctuations in the performance, especially in the creep residual strain performance.

### 5.2. The Temperature Sensitivity of the Dissolution of Primary γ′_I_ Phases

**Figure 15:**
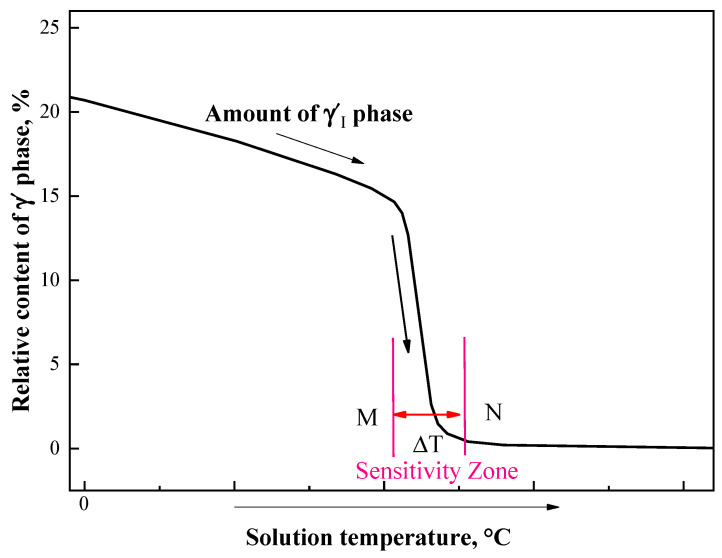
Schematic diagram of the sensitivity zone of the dissolution of primary γ′_I_ phases.

Based on the above analysis of the microstructure evolution and creep residual strain properties after heat treatment at different solution temperatures, it can be found that there is a temperature interval of sensitivity of the γ′_I_ phases near the solution temperature of 1040 °C. The interval M-N shown in Figure 15 is the temperature range ΔT of this sensitivity zone for the γ′_I_ phase dissolution temperature. Generally, this interval is located close to the temperature at which the γ′_I_ phase is completely redissolved.

It is worth mentioning, if the alloy undergoes heat treatment with a solution temperature in the sensitivity zone, it will cause drastic changes to the precipitation and redissolution of the γ′_I_ phases, i.e., it is more sensitive to temperature within that zone. In this sensitivity zone, even a small temperature change will cause a large change in the number of γ′_I_ phases. Because the total amount of γ′ phases depends on the chemical composition, from the perspective of phase quantities, one γ′_I_ phase can be replaced by multiple γ′_II_ phases and γ′_III_ phases. Therefore, when the γ′_I_ phases are dissolved into the matrix, a larger number of γ′_II_ phases and γ′_III_ phases will precipitate, which, in turn, leads to significant fluctuations in the mechanical properties (such as creep properties).

### 5.3. The Effect of Al + Ti Content on γ′ Phases

**Figure 16:**
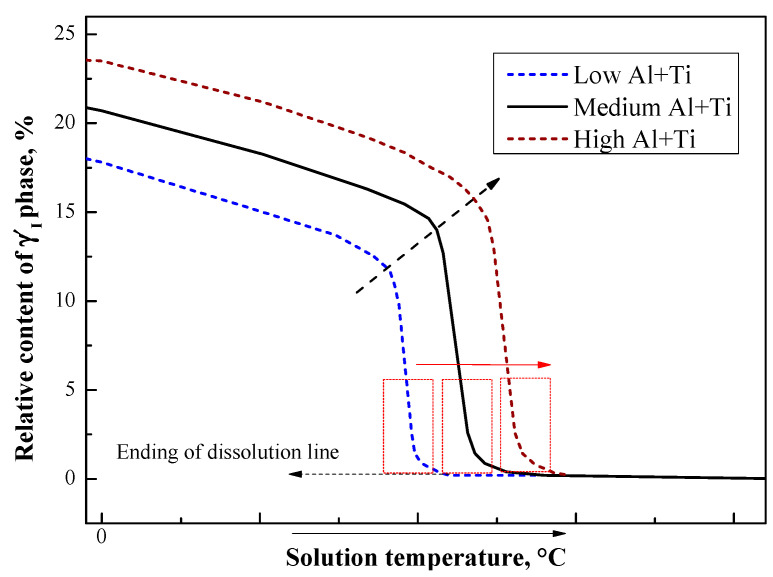
The effect of heat treatment solution temperature and Al + Ti content on the γ′_I_ phase.

The above studies have analyzed the reasons for the creep performance fluctuations caused by the solution heat treatment in the temperature sensitivity zone of the γ′_I_ phase while determining that the temperature range of the sensitivity zone will change with the content of the γ′_I_ phase forming elements (Al and Ti). A schematic diagram (Figure 16) of the relationship between Al + Ti content, solution temperature, and the relative content of the γ′_I_ phase is constructed based on thermodynamic calculation by Thermo-Calc software supported by Sweden and related reports [40,41].

**Figure 17:**
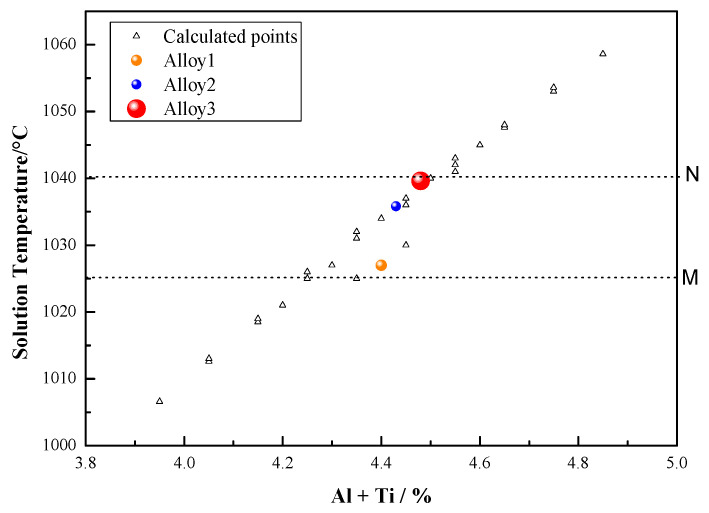
The evolution of the dissolution temperature of the primary γ′_I_ phases with Al + Ti% content, indicating the sensitivity zone for a sample with composition indicated with the open circle.

As shown in Figure 16, the sensitivity zone (indicated by a dotted frame), is close to the dissolution temperature of γ′_I_ phases. As the Al + Ti content increases, the temperature of the sensitivity zone increases, and conversely, when Al + Ti content become lower, its temperature of the γ′_I_ phase-sensitive zone decreases. Therefore, the selection of the heat treatment solution temperature also needs to consider the Al + Ti content of the alloy. Figure 17 shows the evolution of the sensitivity zone of the primary γ′_I_ phase dissolution with Al + Ti% content, from calculation with Thermo-Calc software. The MN sensitivity zone shown is for an alloy with composition indicated with a circle symbol. M indicates the temperature at which the amount of the γ′_I_ phase begins to change rapidly, and N indicates the dissolution temperature above which no primary γ′_I_ phase is observed. With increasing Al + Ti, the calculated dissolution temperature increases (shown with white triangles for various compositions). The different dissolution temperatures of the three experimental alloys are shown with red dots. This explains why the creep residual strain value measured for Alloy3 (high Al + Ti content) does not fluctuate much when it is solution heat treated at 1040 °C. The high Al + Ti content makes the temperature sensitivity zone move up to the high temperature section. This means that the heat treatment solution temperature at 1040 °C is lower than its sensitivity zone. Therefore, there are not large fluctuations in the size and quantity of γ′_I_ phases, and as a result, the creep performance has only small fluctuations.

Since the alloy undergoes solution heat treatment plus double aging, the total amount of γ′ phases remain constant. Therefore, in this experiment, where the solution temperature is around 1040 °C and the Al + Ti content remains unchanged, an increase in the solution temperature will reduce the number of γ′_I_ phases, which will inevitably lead to an increase in the precipitation of γ′_II_ phases or γ′_III_ phases. In addition, because of the effect of the cooling rate on the γ′ phases, when the Al + Ti content and the heat treatment solution temperature are fixed, increasing the cooling rate after solution heat treatment will reduce the number of γ′_I_ phases. The solution temperature, Al + Ti content, and cooling rate all affect the γ′ phase distribution, which can lead to fluctuations and instability in alloy properties.

## 6. Conclusions

In this work, heat treatment experiments with different solution temperatures were carried out for alloys with different Al + Ti contents. The microstructure evolution, creep residual strain, low cycle fatigue properties, and tensile properties were analyzed. The key reason for fluctuation of mechanical properties in nickel-based superalloys was revealed. The research is summarized below:

(1) Following heat treatment with a solution temperature of 1020 °C, there was a high primary γ′_I_ phase content, which is beneficial to maintaining a low creep residual strain. By increasing the cooling rate after solution heat treatment by adopting an oil cooling method, the number of fine precipitate phases increased significantly, which greatly improved the yield strength of the alloy. Following heat treatment with a solution temperature of 1040 °C, there were significant fluctuations in the value of creep residual strain and low cycle fatigue performance.
(2) The essential reason for the dramatic fluctuation of creep performance is the distribution of γ′_I_ phases of different sizes and quantities in the alloy, especially following solution heat treatment in the temperature sensitivity zone of the γ′_I_ phase, which is likely to cause huge fluctuations in number of tertiary γ′_III_ phases. In this morphology regime, the more tertiary γ′_III_ phases, the lower the creep residual strain.
(3) It is proposed that there is a temperature zone for solution heat treatment of particular sensitivity for γ′_I_ phases, and heat treatments performed in this temperature range are likely to cause performance fluctuations. The experimental alloy investigated has this temperature sensitivity around 1040 °C.
(4) The solution temperature to achieve stable properties should be adjusted according to the Al + Ti content of the alloy. An increase in the γ′ content will increase the dissolution temperature of γ′ phases. In order to keep the mechanical properties stable and avoid large fluctuations, the influence of the temperature-sensitive zone for the γ′ phase at the specific Al + Ti content should be considered.

## Figures and Tables

**Table 1:** The composition of three alloys investigated (wt%).

Elements	C	Cr	Mo	Ti	Al	Co	Zr	B	Al + Ti	Ni
Alloy1	0.04	19.36	4.34	3.03	1.36	13.55	0.052	0.0054	4.39	Balance
Alloy2	0.04	19.39	4.32	3.04	1.39	13.52	0.056	0.0048	4.43	Balance
Alloy3	0.05	19.37	4.32	3.10	1.38	13.69	0.046	0.0043	4.48	Balance

**Table 2:** The tensile yield strength of alloys at room temperature (Unit: MPa).

Heat Treatment	Al + Ti, wt%	1020 °C/4 h/AC + 845 °C/4 h/AC + 760 °C/16 h/AC	1020 °C/4 h/OC + 845 °C/4 h/AC + 760 °C/16 h/AC
Alloy1	4.39	780.1	909.3
Alloy2	4.43	769.9	924.6
Alloy3	4.48	810.2	960.0

## Data Availability

The processed data required to reproduce these findings can be shared.
